# Scavenger receptor collectin placenta 1 is a novel receptor involved in the uptake of myelin by phagocytes

**DOI:** 10.1038/srep44794

**Published:** 2017-03-20

**Authors:** Jeroen F. J. Bogie, Jo Mailleux, Elien Wouters, Winde Jorissen, Elien Grajchen, Jasmine Vanmol, Kristiaan Wouters, Niels Hellings, Jack Van Horsen, Tim Vanmierlo, Jerome J. A. Hendriks

**Affiliations:** 1 Biomedical Research Institute, Hasselt University/Transnational University Limburg, School of Life Sciences, Diepenbeek, Belgium; 2 Cardiovascular Research Institute Maastricht (CARIM), Maastricht University Medical Centre (MUMC), Maastricht, The Netherlands; 3 Department of Internal Medicine, Maastricht University Medical Centre (MUMC), Maastricht, The Netherlands; 4 Department of Molecular Cell Biology and Immunology, VU University Medical Center, Amsterdam, The Netherlands

## Abstract

Myelin-containing macrophages and microglia are the most abundant immune cells in active multiple sclerosis (MS) lesions. Our recent transcriptomic analysis demonstrated that collectin placenta 1 (CL-P1) is one of the most potently induced genes in macrophages after uptake of myelin. CL-P1 is a type II transmembrane protein with both a collagen-like and carbohydrate recognition domain, which plays a key role in host defense. In this study we sought to determine the dynamics of CL-P1 expression on myelin-containing phagocytes and define the role that it plays in MS lesion development. We show that myelin uptake increases the cell surface expression of CL-P1 by mouse and human macrophages, but not by primary mouse microglia *in vitro*. In active demyelinating MS lesions, CL-P1 immunoreactivity was localized to perivascular and parenchymal myelin-laden phagocytes. Finally, we demonstrate that CL-P1 is involved in myelin internalization as knockdown of CL-P1 markedly reduced myelin uptake. Collectively, our data indicate that CL-P1 is a novel receptor involved in myelin uptake by phagocytes and likely plays a role in MS lesion development.

Multiple sclerosis (MS) is a chronic, inflammatory, neurodegenerative disease of the central nervous system (CNS). Macrophage- and microglia-mediated myelin destruction is considered to be the primary effector mechanism in MS lesion development^1^. Previous studies defined that complement-receptor 3, scavenger receptors I/II, and Fcγ receptors, facilitate the clearance of myelin by macrophages and microglia^2^,^3^. However, considering the complexity of myelin, it is unlikely that solely these receptors are involved in the uptake of myelin by activated microglia and macrophages in MS lesions.

Using genome wide gene expression analysis, we previously found that internalization of myelin alters the expression of 676 genes in rat peritoneal macrophages^4^. Collectin placenta 1 (CL-P1) was one of the most potently induced genes in macrophages upon uptake of myelin. CL-P1 is structurally related to scavenger receptor class A (SRA) due to its collagen-like domain^5^. However, CL-P1 also contains a C-type lectin/carbohydrate recognition domain (C-type CRD)^6^,^7^, typically found in C-type lectin receptors, such as dendritic cell-specific ICAM3-grabbing non-integrin (DC-SIGN)^8^. Functionally, CL-P1 is associated with binding and internalization of bacteria, yeast, and oxidized low-density lipoproteins^5^,^6^,^7^,^9^. Furthermore, CL-P1 recognizes carcinoma-associated antigens, possibly via interaction with Lewis^x^ trisaccharide on tumor cells^10^,^11^, hereby mediating tumor cell-endothelium interactions^12^,^13^. Finally, a recent study showed that the collagen-like domain of CL-P1 facilitates amyloid beta (Aβ) clearance by microglia and that uptake of Aβ increases the expression of CL-P1^14^. These findings indicate that CL-P1 plays a role host defense and cellular uptake in different diseases.

In this study, we sought to determine if myelin internalization increases surface expression of CL-P1 on peripheral and CNS-resident phagocytes, its involvement in internalization of myelin, and its cellular distribution in MS lesions. We show that myelin uptake increases the cell surface expression of CL-P1 by mouse and human macrophages, but not by primary mouse microglia *in vitro*. In active MS lesions CL-P1 immunoreactivity was localized to parenchymal and pervivascular myelin-containing phagocytes. Finally, we show that silencing of CL-P1 strongly reduces myelin uptake. Collectively, our data indicate that CL-P1 mediates the uptake of myelin and likely plays a role in MS lesion development.

## Results

### Myelin increases the surface expression of CL-P1 on phagocytes

**Figure 1:**
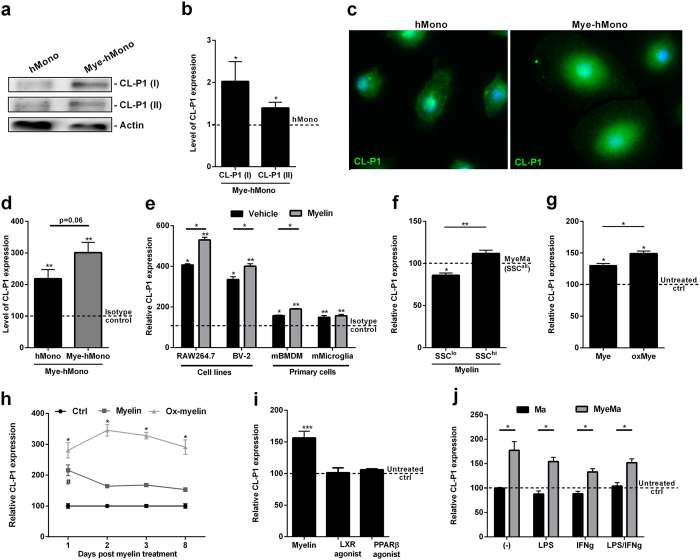
Myelin uptake increases the surface expression of CL-P1 on myeloid cells. (**a,b**) Human monocytes (hMono, n = 5), were cultured with or without 100 μg/ml myelin for 24 h. Western blot analysis was used to define CL-P1 expression. Two antibodies were used to define CL-P1 expression. Western blots are displayed in cropped format. (**c**) Immunohistochemistry (CL-P1, Novus Biologicals) was used to define the expression of CL-P1 by human monocytes cultured with or without 100 μg/ml myelin for 24 h. (**d**) Human monocytes (n = 7) were cultured with or without 100 μg/ml myelin for 24 h. CL-P1 expression was determined with flow cytometry (CL-P1, R&D). Dotted line represents untreated cells stained with an isotype antibody. (**e**) RAW264.7 (n = 4), BV-2 (n = 4), mouse BMDMs (n = 4), and mouse microglia (n = 7), were cultured with or without 100 μg/ml myelin for 24 h. CL-P1 expression was determined with flow cytometry (CL-P1, R&D). Dotted line represents untreated cells stained with an isotype control antibody. (**f**) Mouse BMDMs cultured with 100 μg/ml myelin for 24 h. CL-P1 expression was determined in high granular (SSC^hi^), low granular (SSC^lo^), and all cells (SSC^all^) using flow cytometry. Dotted line represents myelin-treated cells stained with the CL-P1 antibody (n = 4). (**g**) RAW264.7 cells were exposed to 100 μg/ml unmodified and CU^2^-oxidized myelin for 24 h, after which CL-P1 expression was determined. Dotted line represents untreated cells stained with the CL-P1 antibody (n = 4). (**h**) RAW264.7 cells were cultured with 100 μg/ml unmodified or CU^2^-oxidized myelin for 1,2,3, and 8 days (n = 3). CL-P1 expression was determined by using flow cytometry. (**i**) RAW264.7 cells were cultured with a T0901317 (LXR agonist), GW501516 (PPARβ/δ agonist), or 100 μg/ml myelin for 24 h. CL-P1 expression was determined with flow cytometry. Dotted line represents untreated cells stained with the CL-P1 antibody (n = 6). (**j**) Untreated or myelin treated RAW264.7 cells were exposed to 500 U/ml IFNγ, 100 ng/ml LPS, a combination IFNγ and LPS, or left untreated (n = 4). CL-P1 expression was determined using flow cytometry. Dotted line represents untreated cells stained with the CL-P1 antibody. Data are presented as mean ± SEM. *p < 0.05, **p < 0.01, ***p < 0.001.

By using a transcriptomic approach, we previously demonstrated that myelin induces gene expression of CL-P1 in peritoneal rat macrophages^4^. Here, we validated this increase in CL-P1 mRNA expression on protein level in mouse and human primary phagocytes and phagocyte cell lines. By using western blot (Fig. 1a,b and s1), immunohistochemistry (Fig. 1c), and flow cytometry (Fig. 1d and s2a), we show that human primary monocytes express CL-P1 and that myelin internalization increases the expression of CL-P1. For western blot analysis, two separate antibodies were used to confirm the myelin-induced increase in CL-P1 expression. We further show that mouse primary microglia and bone-marrow derived macrophages (BMDMs), as well as cell lines closely resembling these phagocytes (BV-2, microglia; RAW264.7, macrophages), express CL-P1 and that myelin uptake results in an elevated expression of CL-P1 by these cells (Fig. 1e and s2b–e). Interestingly, CL-P1 expression was not increased on primary mouse microglia after myelin uptake. In addition, we found increased expression of CL-P1 by high granular (SSC^hi^) myelin-containing mouse primary BMDMs compared to low granular (SSC^lo^) cells that did not substantially phagocytose myelin (Fig. 1f and s2f). This finding indicates that the intensity of CL-P1 immunoreactivity correlates with the amount of internalized myelin.

In MS lesions, phagocytes are likely to encounter modified forms of myelin such as oxidized myelin^15^,^16^,^17^. We demonstrate that oxidized myelin more prominently increases the surface expression of CL-P1 on macrophages compared to unmodified myelin (Fig. 1g and s2g). In addition, while CL-P1 surface expression gradually decreased on macrophages treated with unmodified myelin, macrophages treated with oxidized myelin retained a high expression of CL-P1 over time (Fig. 1h and s2h–k).

Previously, we found that myelin-derived lipids, such as cholesterol metabolites and fatty acids, partially account for the phenotype of phagocytes after myelin uptake^4^,^18^. Activation of the liver X receptor (LXR) and peroxisome proliferator-activated receptor β/δ (PPARβ/δ) underlies the impact of these lipids on the phenotype of phagocytes. By using synthetic agonists for LXR and PPARβ/δ, we show that myelin increases the expression of CL-P1 in an LXR- and PPARβ/δ-independent manner (Fig. 1i and s2l). We further demonstrate that inflammatory stimuli, such as IFNγ and LPS, do not impact CL-P1 expression by both untreated and myelin-treated macrophages (Fig. 1j and s2m,n). Collectively, these data show that myelin uptake increases the surface expression of CL-P1 on phagocytes *in vitro* in an LXR- and PPARβ/δ-independent manner, and that inflammatory stimuli do not impact CL-P1 expression.

### CL-P1 is expressed by phagocytes in MS lesions

**Figure 2:**
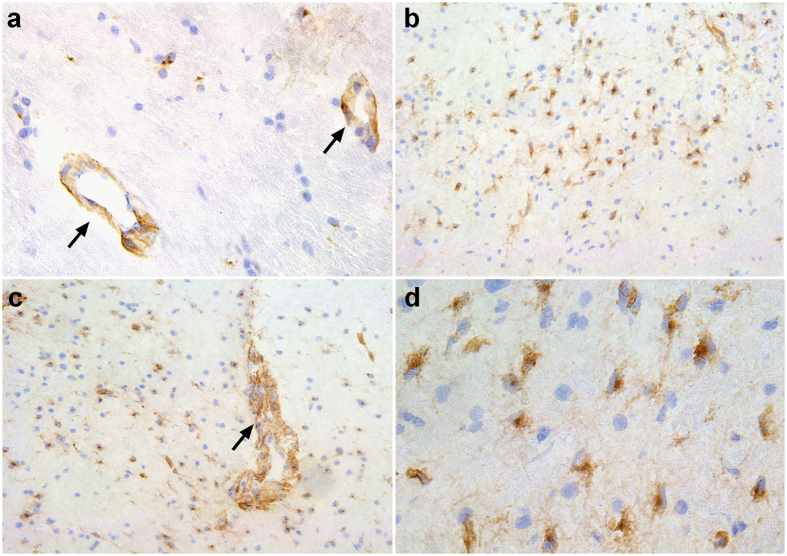
CL-P1 is highly expressed in MS lesions. (**a**) Image of normal-appearing matter stained for CL-P1 (40x magnification). Arrows depict blood vessels. (**b–d**) Active MS lesion stained for CL-P1 (**b,c**, 10x magnification; **d**, 40x magnification). Arrow depicts a perivascular cuff filled with infiltrated myeloid cells.

**Figure 3:**
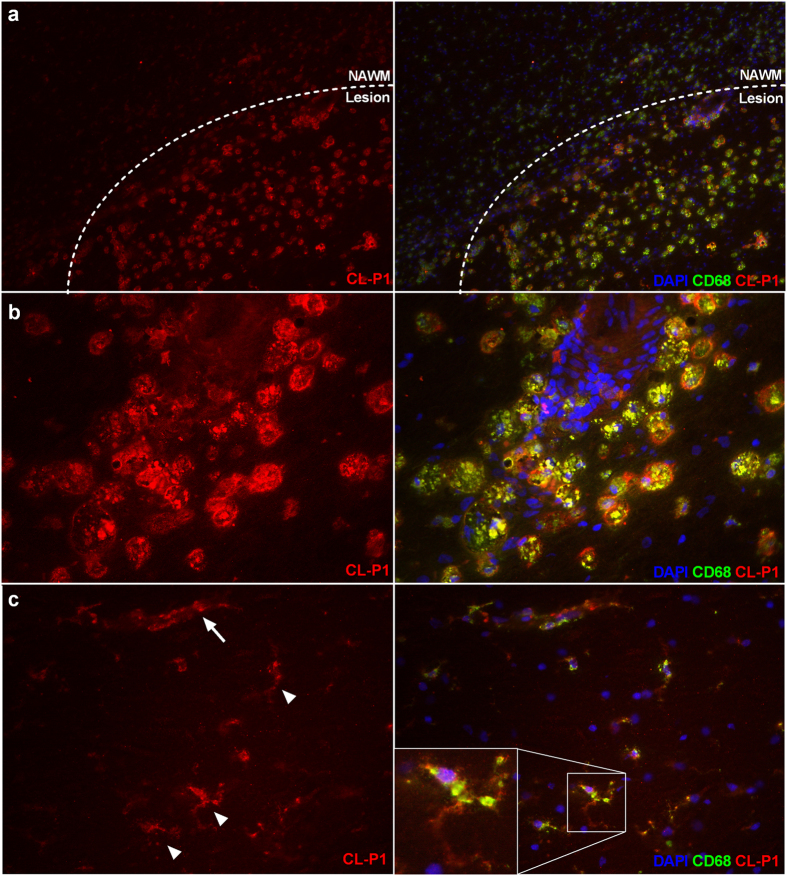
CL-P1 is expressed by phagocytes in MS lesions. (**a,b**) Representative images of active MS lesion stained for CD68 and CL-P1 (Novus Biologicals; (**a**), 10x magnification; (**b**), 40x magnification). (**c)** NAWM stained for CD68 and CL-P1 (Novus Biologicals, 40x magnification). Perivascular macrophages and microglia are designated by an arrow and arrowheads, respectively.

**Figure 4:**
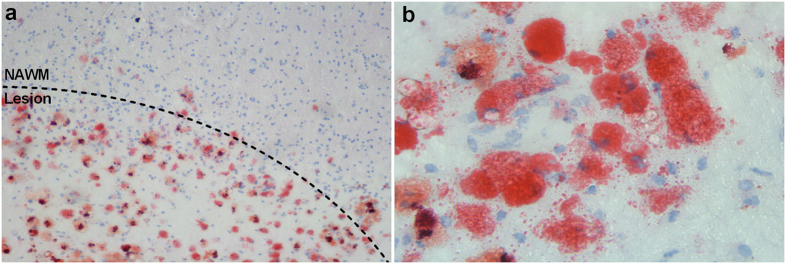
Abundant lipid-containing phagocytes in perivascular space and parenchyma of active MS lesion. (**a,b**) ORO staining of active MS lesion showing foamy phagocytes containing neutral intracellular lipids (**a**, 10x magnification; **b**, 40x magnification).

The observed increase in the expression of CL-P1 on macrophages following myelin internalization *in vitro*, prompted us to determine CL-P1 expression in active MS lesions. We show that CL-P1 is predominantly expressed on brain endothelial cells in the normal-appearing white matter (NAWM) (Fig. 2a). In MS lesions, a profound increase in the expression of CL-P1 was observed (Fig. 2b–d). Immune-double labeling revealed that CD68^+^ parenchymal and perivascular phagocytes expressed CL-P1 within MS lesions (Fig. 3a,b). Within the NAWM, CD68^+^ microglia and perivascular macrophages expressed CL-P1 (Fig. 3c). Findings were validated using an alternative antibody directed against CL-P1 (Fig. s3a,b). Interestingly, within active MS lesions, GFAP^+^ astrocytes also expressed CL-P1 (Fig. s3c). Control staining did not show any immunoreactivity (data not shown). Oil Red O staining further showed that lipid-containing phagocytes were abundantly present in both the parenchyma and perivascular spaces within these lesions (Fig. 4a,b). These data indicate that CL-P1 is expressed on astrocytes and myelin-laden perivascular and parenchymal phagocytes within active MS lesions.

### CL-P1 mediates the uptake of myelin

**Figure 5:**
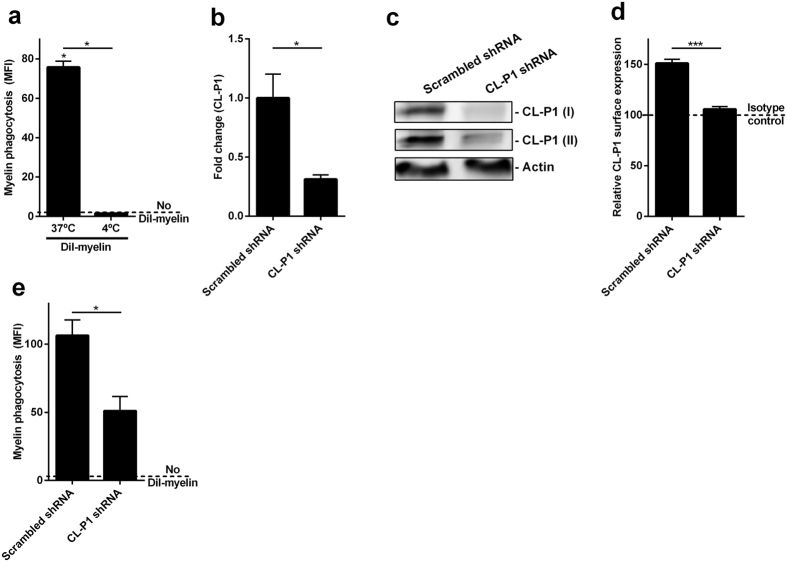
CL-P1 is involved in the uptake of myelin. (**a**) HEK293.1 cells were exposed to DiI-labeled myelin for 1.5 h (n = 4). Myelin uptake was assessed using flow cytometry. Cells were exposed to myelin at 4*°*C (binding) or 37 °C (binding and uptake). Dotted line represents untreated cells. (**b–d**) HEK293.1 cells were exposed to scrambled shRNA or a pool of shRNA directed against CL-P1 (shRNA1-4) for 48 h. The mRNA and protein expression of CL-P1 was determined using qPCR (**b**, n = 4), western blot (c, CL-P1 I (R&D), CL-P1 II (Novus Biologicals), n = 3), and flow cytometry (**d**, n = 6). Western blots are displayed in cropped format. (**e**) HEK293.1 cells were exposed to scrambled shRNA or a pool of shRNA directed against CL-P1 (shRNA1-4) for 48 h. Next, DiI-labeled myelin was added for 1.5 h (n = 8). Flow cytometry was used to define myelin uptake. Dotted line represents untreated cells. Data are presented as mean ± SEM. *p < 0.05, **p < 0.01, ***p < 0.001.

Considering that CL-P1 is structurally related to SRA and that the uptake of myelin by phagocytes is mediated by SRA^3^,^5^, we determined whether CL-P1 is involved in the internalization of myelin. For this purpose, plasmids expressing shRNA directed against CL-P1 were used. HEK293.1 cells were used as an easy transfectable human cell line with phagocytic properties. Importantly, HEK293.1 avidly endocytosed human myelin debris (Fig. 5a and s4a) and expressed CL-P1 (Fig. 5b–d). To define the knockdown efficacy and the role that CL-P1 plays in the uptake of myelin, HEK293.1 cells were exposed to a pool of shRNAs directed against CL-P1. We show that the pool of shRNAs (shRNA1–4) completely reduced the cell surface expression of CL-P1 compared to scrambled shRNA (Fig. 5e and s4b). Western blot and qPCR analysis demonstrated a ~60% reduction in CL-P1 expression when cells were exposed to Cl-P1 shRNAs (Fig. 5c,d and s5). Importantly, we show that silencing of CL-P1 reduced the uptake of myelin by ~50% compared to scrambled shRNA (Fig. 5e and s4c). These data indicate that CL-P1 is involved in the internalization of myelin.

## Discussion

Foamy phagocytes containing myelin debris are the most abundant immune cells in active MS lesions. Our recent transcriptomic analysis demonstrated that CL-P1 is one of the most potently induced genes in macrophages after uptake of myelin. In this study we sought to determine the dynamics of CL-P1 expression on myelin-phagocytosing phagocytes and unravel what function CL-P1 has on these phagocytes. We show that CL-P1 is expressed by phagocytes in inflammatory MS lesions and that myelin uptake induces cell surface expression of CL-P1 in mouse and human phagocytes *in vitro*. Moreover, we demonstrate that CL-P1 is involved in myelin internalization as knockdown of CL-P1 markedly reduced myelin uptake. These data indicate that CL-P1 is a novel receptor involved in the internalization of myelin by macrophages and likely plays a role in the pathophysiology of MS.

In this study, we show that both mouse macrophages and human monocytes express CL-P1 on their cell surface and that myelin internalization increases the surface expression of CL-P1 on BMDMs in a dose-dependent manner *in vitro*. However, whereas primary mouse microglia expressed CL-P1, myelin internalization did not increase the expression of CL-P1 by these phagocytes. This discrepancy may underline the fact that microglia and infiltrating macrophages react differently to environmental cues^19^,^20^,^21^. Ontogenic differences in signaling pathways involved in the regulation of CL-P1 might explain the observed discrepancy between the two phagocyte subsets^22^,^23^. In active MS lesions, HLA-DR^+^ phagocytes markedly expressed CL-P1 suggesting that myelin internalization also enhances CL-P1 expression by phagocytes in MS lesions.

Myelin is composed of a variety of lipids and proteins, many of which can alter the physiology of phagocytes upon binding and internalization. Recently, we showed that myelin uptake skews macrophages towards a less-inflammatory phenotype, at least in part, through the activation of the lipid sensing LXR and PPAR^4^,^18^. Unlike SRAs, such as SPα, MARCO, and CD36, which are well-known target genes of LXRs or PPARs^24^,^25^, we found that the expression of CL-P1 was not regulated by agonists for either of these nuclear receptors. Likewise, inflammatory signaling pathways activated by IFNγ and LPS did not significantly impact the surface expression of CL-P1 on control and myelin-containing phagocytes *in vitro*. Future studies are needed to elucidate how myelin uptake regulates the expression of CL-P1. Interestingly, oxidized myelin more potently induced and maintained the expression of CL-P1 on phagocytes compared to unmodified myelin. Defining transcriptional differences between phagocytes exposed to unmodified and oxidized myelin may lead to the identification of the biological pathway controlling CL-P1 expression.

Several receptors, such as the complement-receptor 3, SRA I/II, and Fcγ receptors, facilitate the clearance of myelin by macrophages and microglia^2^,^3^. Our data indicate that CL-P1 also contributes to the internalization of myelin. The phagocytic capacity of SRA largely depends on its collagen-like domain^26^. Considering that CL-P1 and SRA share the same collagen-like domain^5^, this domain may underlie the role that CL-P1 plays in the internalization of myelin. Future studies are warranted to determine if CL-P1 contributes to myelin uptake *in vivo* and how this impacts neuroinflammation and neurodegeneration. As uptake of myelin leads to both demyelination and CNS repair, depending on whether it concerns intact myelin or myelin debris, CL-P1-mediated myelin uptake can be both beneficial or detrimental^1^,^27^,^28^,^29^. In our *in vitro* experiments, myelin debris is used to define the impact of CL-P1 on the uptake of myelin. Hence, it is tempting to speculate that CL-P1 might play a role in myelin debris clearance *in vivo*, thereby facilitating remyelination^27^,^28^,^29^.

Aside from a collagen-like domain, CL-P1 contains a C-type CRD that binds with high affinity to glycans bearing Lewis^x^ and Lewis^a^ trisaccharides^10^,^11^. Interestingly, based on this glycan-specificity, parallels can be drawn between CL-P1 and both DC-SIGN and selectins^30^,^31^. This suggests that CL-P1 may also play a role in cell migration, cell differentiation, antigen-capture, and T cell priming^32^,^33^. Interestingly, we found that CL-P1 is markedly expressed on foamy-appearing phagocytes in and near perivascular cuffs in MS lesions. As perivascular cuffs accommodate lymphocytes during active MS, CL-P1 on phagocytes may play a role in T cell priming. Additionally, as myelin-containing phagocytes are located in CNS-draining lymphoid organs^34^,^35^,^36^, future studies should determine whether CL-P1 may facilitate lymph node directed migration of these phagocytes.

Increasing evidence indicates that astrocytes actively participate in various processes underlying MS pathogenesis, including neuroinflammation, demyelination, and remyelination^37^. We show that astrocytes have increased expression of CL-P1 in MS lesions. Of interest, CL-P1 immunoreactivity is also increased on reactive astrocytes in AD^14^. Follow-up studies should address whether this increased expression of CL-P1 on astrocytes in MS lesions plays a role in the phagocytic capacity of astrocytes, as well as their migration and differentiation.

Based on our findings, we propose a positive feedback model in which CL-P1 mediates the uptake of myelin by phagocytes and subsequently increases its own expression. Considering its role in the uptake of myelin, CL-P1 likely plays an important role in the pathophysiology of MS.

## Methods

### Cell isolation and culture

Bone marrow-derived macrophages were obtained as described previously^38^. Briefly, femoral and tibial bone marrow suspensions from 12 week-old C57Bl/6J mice (Harlan, Horst, Netherlands) were cultured in 10 cm plates at a concentration of 10 × 10^6^ cells/plate and differentiated in RPMI 1640 medium (Invitrogen, Merelbeke, Belgium) supplemented with 10% fetal calf serum (FCS, Gibco, Merelbeke, Belgium), 50 U/ml penicillin (Invitrogen), 50 U/ml streptomycin (Invitrogen), and 15% L929-conditioned medium. Microglia cultures were prepared from postnatal P3 C57BL/6J mouse pups. Isolated forebrains of mice pups were placed in L15 Leibovitz medium (Gibco) containing 1:10 Trypsin (Sigma-Aldrich, Diegem, Belgium) (37 °C, 15 min). Next, high glucose DMEM medium (Invitrogen) supplemented with 10% FCS, 50 U/ml penicillin, 50 U/ml streptomycin, (DMEM 10:1 medium), and 100 μl/ml DNase I (Sigma-Aldrich) was added to the forebrain tissue. Nervous tissue was dissociated by trituration with serum-coated Pasteur pipettes (Sigma-Aldrich). The dissociated mix was passed through a 70 μm cell strainer, rinsed with 5 ml of DMEM 10:1 medium, and centrifuged (170 g, 10 min, RT). After a second centrifugation step, cell suspension was seeded at 2 forebrains/75 cm^2^ flask. After 2 days, DMEM 10:1 medium was changed and after reaching confluence (±6 days later), 2/3 DMEM 10:1 medium containing 1/3 L929-conditioned medium was added. Six days later, microglia isolation was performed using the shake-off method (200 rpm, 2 h, RT). Microglia were centrifuged (170 g, 10 min, RT), suspended in DMEM 10:1 medium containing B27 supplement (Invitrogen), and cultured at 250.000 cells/well in poly-L-lysine (Sigma-Aldrich)-coated 24-well plates. Animals were housed in the animal facility of the Biomedical Research Institute of Hasselt University. All experimental protocols and methods involving animals within this study were conducted in accordance with institutional guidelines and approved by the Ethical Committee for Animal Experiments Hasselt University.

Peripheral blood mononuclear cells were isolated from whole blood by density gradient centrifugation on lympholyte-H cell separation media (Cedarlane, Ontario, Canada). Blood samples were collected from healthy controls after obtaining informed written consent. Subjects with signs of infection were excluded. All experimental protocols and methods were conducted in accordance with institutional guidelines and approved by the Medical Ethical Committee Hasselt University. CD14^+^ monocytes were collected using the EasySep human CD14 positive selection kit (Stemcell Technologies, Grenoble, France) according to manufacturer’s instructions. After isolation, cells were cultured (1 × 10^6^ cells/ml) in RPMI 1640 supplemented with 10% human serum (Sigma-Aldrich, Saint Louis, USA), 50 U/ml penicillin and 50 U/ml streptomycin.

The immortalized mouse macrophage (RAW 264.7), mouse microglia (BV-2), and human embryonic kidney (HEK293.1) cell lines were cultured in DMEM (Invitrogen) with 50 U/ml penicillin, 50 U/ml streptomycin), and 10% FCS. To determine the effect of myelin and LXR and PPARβ/δ agonists for LXR and PPARβ/δ on the expression of CL-P1, cells were treated for 24 hours with 100 μg/ml of isolated myelin, 10 μM T0901317 (T09; LXR agonist; Cayman Chemical, Huissen, The Netherlands), or 10 μM GSK0660 (PPARβ/δ agonist; Sigma-Aldrich). To determine the impact of inflammation on CL-P1 expression, cells exposed to 100 ng/ml LPS (Sigma-Aldrich) and/or IFNγ (Peprotech, Hamburg, Germany).

### Myelin isolation, labelling, and phagocytosis

Myelin was purified from postmortem mouse and human brain tissue by means of density gradient centrifugation, as described previously^39^. Experimental protocols and methods were conducted in accordance with institutional guidelines and approved by the Medical Ethical Committee Hasselt University and the Ethical Committee for Animal Experiments Hasselt University. Written informed consent was obtained from all donors. Myelin protein concentration was determined by using the BCA protein assay kit (Thermo Fisher Scientific, Erembodegem, Belgium), according to manufacturer’s instructions. Endotoxin content was determined using the Chromogenic Limulus Amebocyte Lysate assay kit (Genscript Incorperation, Aachen, Germany). Isolated myelin contained a negligible amount of endotoxin ( ≤ 1.8 × 10^−3^ pg/μg myelin). To obtain oxidized myelin, myelin was exposed to 10 μM CuSO4 at 37 °C for 20 hours. Myelin was fluorescently labelled, according to the method of Van der Laan *et al*.^40^. In short, 10 mg/ml myelin was incubated with 12.5 μg/ml 1,1”-diotadecyl-3,3,3′,3′,-tetramethylindocarbocyanide perchlorate (DiI; Sigma-Aldrich) for 30 min at 37 °C. To determine the capacity of cells to phagocytose myelin, cells were exposed to 100 μg/ml DiI-labeled myelin. The amount of myelin phagocytosed was determined using a FACSCalibur (BD Biosciences, Erembodegem, Belgium). HEK293.1 were used to define the impact of CL-P1 on myelin phagocytosis as BV-2 and RAW264.7 cells are not easily transfectable. Of note, HEK293.1 are often used as a model system to study phagocytic receptors^41^,^42^.

### Western blot

CL-P1 protein expression was determined via SDS-PAGE and western blot analysis. Briefly, samples were denaturated and separated on a 8% polyacrylamide gel containing Tris-glycine and transferred onto a polyvinylidene difluoride (PVDF) membrane (GE Healthcare, Buckinghamshore, UK). Non-specific binding was blocked by incubating the membranes in 5% (w/v) nonfat powdered milk in Tris-buffered saline containing 0.1% (v/v) Tween-20 (TBS-T) for 1 hour. Subsequently, membranes were incubated with primary antibodies goat anti-human CL-P1 (R&D Systems, Abingdon, UK 1:1000), goat-anti-human CL-P1 (Novus Biologicals, Abingdon, UK, 1:1000), and rabbit anti-human B-actin (1:10000, Santa Cruz Biotechnology, Heidelberg, Germany) in TBS-T overnight at 4 °C. Membranes were incubated for 1 hour at room temperature with a horseradish peroxidase-conjugated rabbit-anti goat and goat anti-rabbit antibodies (Dako, 1:2000) in 5% milk in TBS-T. For stripping and reprobing, a mild stripping buffer was used (0.2 M glycine, 0.1% SDS, 1% Tween-20, pH 2.2). An ECL Plus detection kit (Thermo Fisher Scientific) was used and the generated chemiluminescent signal was detected by a luminescent image analyzer (ImageQuant LAS 4000 mini; GE Healthcare).

### shRNA and transfection

The X-tremegene HP transfection kit (Roche Diagnostics, Mannheim, Germany) was used to transfect HEK293.1 cells according to the manufacturer’s instructions. In short, 0.25 × 10^6^ HEK293.1 cells were transfected with 1.5 μg of shRNA in 50 μl Opti-MEM^®^ I Reduced Serum Media (Thermo Fisher Scientific). Cells were then resuspended in complete culture medium and incubated for 48 hours at 37 °C. CL-P1 (shRNA-1); AACATCTCGCCAAACCTATGA, CL-P1 (shRNA-2); CAGGCTATCCAGCGAATCAAGAA, CL-P1 (shRNA-3); AAGAAATGAAGCTAGTAGACT, CL-P1 (shRNA-4); AACGATTTCCAATGTGA AGAC, scrambled; CCTAAGGTTAAGTCGCCCTCG.

### Flow cytometry

Flow cytometry was used to assess the expression of CL-P1 on all cell types. Cells were stained with goat-anti-mouse CL-P1 (R&D Systems), goat-anti-human CL-P1 (R&D Systems), or normal goat IgG (R&D Systems). Alexa fluor 488 F(ab’)_2_ fragment of rabbit-anti goat (Invitrogen) was used as a secondary antibody. The FACSCalibur was used to quantify cellular fluorescence.

### Immunohistochemistry

Frozen brain material from active MS lesions was obtained from the Netherlands Brain Bank (NBB, Amsterdam, Netherlands). Human monocytes were cultured on glass cover slides (Thermo Fisher Scientific) and fixed in 4% PFA for 30 minutes. Cryosections were fixed in acetone for 10 minutes. Cryosections and human monocytes were blocked for 20 minutes with 10% normal serum from the same species as the secondary antibody (Dako, Heverlee, Belgium). For 3, 3′ diaminobenzidine (DAB) staining, slides were incubated with goat-anti-human CL-P1 (R&D Systems). After washing, HRP-conjugated rabbit-anti-goat (Dako) was added. Subsequently, DAB substrate (Dako) was used to stain slides. Sections were counterstained with hematoxylin (Merck, Darmstadt, Germany). For fluorescence staining, cryosections were incubated with goat-anti-human CL-P1 (R&D Systems), goat-anti-human CL-P1 (Novus Biologicals), mouse-anti-human CD68 (Ebioscience, Vienna, Austria), mouse-anti-human Human Leucocyte Antigen DR/DP/DQ (HLA-DR/DP/DQ; Dako), or rabbit-anti glial fibrillary acidic protein (GFAP; Dako). Cryosections were stained with Alexa flour secondary antibodies (Invitrogen). Nuclei were visualized using 4,6′-diamidino-2-phenylindole (DAPI; Invitrogen). Analysis was carried out using a Nikon eclipse 80i microscope and NIS Elements BR 3.10 software (Nikon, Tokyo, Japan). Intracellular myelin degradation products were defined with oil-red O (ORO), which stains neutral lipids, as described previously^43^.

### Quantitative PCR

Total RNA from cultures was prepared using the RNeasy mini kit (Qiagen, Venlo, The Netherlands), according to manufacturer’s instructions. The RNA quality was determined with a NanoDrop spectrophotometer (Isogen Life Science, IJsselstein, The Netherlands). RNA was converted to cDNA using the reverse transcription system (Quanta Biosciences, Gaithersburg, USA) and quantitative PCR was performed on a StepOnePlus detection system (Applied Biosystems, Gaasbeek, Belgium), as previously described^4^,^44^. Relative quantification of gene expression was accomplished using the comparative C_t_ method. Data were normalized to the most stable reference genes^45^,^46^. Primers: *CL-P1* (fw); TGGTAGGGAGAGAGAGCCAC, *CL-P1* (rv); CCCATCCAGCCACTTCCATT, cyclophilin A (*Cyca)* (fv); AGACTGAGTGGTTGGATGGC, *Cyca* (rv); TCGAGTTGTCCACAGTCAGC, ribosomal protein L13A (*Rpl13a)* (fv); AAGTTGAAGTACCTGGCTTTCC, *Rpl13a* (rv); GCCGTCAAACACCTTGAGAC.

### Statistical analysis

Data were statistically analyzed using GraphPad Prism for windows (version 4.03) and are reported as mean ± SEM. D’Agostino and Pearson omnibus normality test was used to test normal distribution. An analysis of variances (ANOVA) or two-tailed unpaired student T-test (with Welch’s correction if necessary) was used for normally distributed data sets. The Kruskal-Wallis or Mann-Whitney analysis was used for data sets which did not pass normality. *P ≤ 0.05, **P ≤ 0.01 and ***P ≤ 0.001.

## Additional Information

**How to cite this article:** Bogie, J. *et al*. Scavenger receptor collectin placenta 1 is a novel receptor involved in the uptake of myelin by phagocytes. *Sci. Rep.* **7**, 44794; doi: 10.1038/srep44794 (2017).

**Publisher's note:** Springer Nature remains neutral with regard to jurisdictional claims in published maps and institutional affiliations.

## Supplementary Material

Supplementary Dataset File
