# Corrigendum: Scavenger receptor collectin placenta 1 is a novel receptor involved in the uptake of myelin by phagocytes

**DOI:** 10.1038/srep46925

**Published:** 2017-12-22

**Authors:** Jeroen F. J. Bogie, Jo Mailleux, Elien Wouters, Winde Jorissen, Elien Grajchen, Jasmine Vanmol, Kristiaan Wouters, Niels Hellings, Jack van Horssen, Tim Vanmierlo, Jerome J. A. Hendriks

Scientific Reports 7: Article number: 44794; 10.1038/srep44794 published online: 03 20 2017; updated: 12 22 2017.

The original version of this Article contained a typographical error in the spelling of the author Jack van Horssen, which was incorrectly given as Jack Van Horsen. This has now been corrected in the PDF and HTML versions of the Article, and in the accompanying Supplementary Dataset File.

