# Supplementary material for: Scavenger receptor collectin placenta 1 is a novel receptor involved in the uptake of myelin by phagocytes
Source: Sci Rep. 2017 Mar 20;7:44794. doi: 10.1038/srep44794 (PMC5357964; doi:10.1038/srep44794)
Supplement: Supplementary Dataset File [file srep44794-s1.pdf]

Supplementary figures

Scavenger receptor collectin placenta 1 is a novel receptor involved in the uptake of myelin by phagocytes

Jeroen Bogie[1,+], Jo Mailleux[1,+], Elien Wouters[1], Winde Jorissen[1], Elien Grajchen[1], Jasmine Vanmol[1], Kristiaan Wouters[2,3], Niels Hellings[1], Jack Van Horsen[4], Tim Vanmierlo[1], Jerome Hendriks[1*]

[1] Biomedical Research Institute, Hasselt University / Transnational University Limburg, School of Life Sciences, Diepenbeek, Belgium

[2] Cardiovascular Research Institute Maastricht (CARIM), Maastricht University Medical Centre (MUMC), Maastricht, The Netherlands

[3] Department of Internal Medicine, Maastricht University Medical Centre (MUMC), Maastricht, The Netherlands

[4] Department of Molecular Cell Biology and Immunology, VU University Medical Center, Amsterdam, The Netherlands

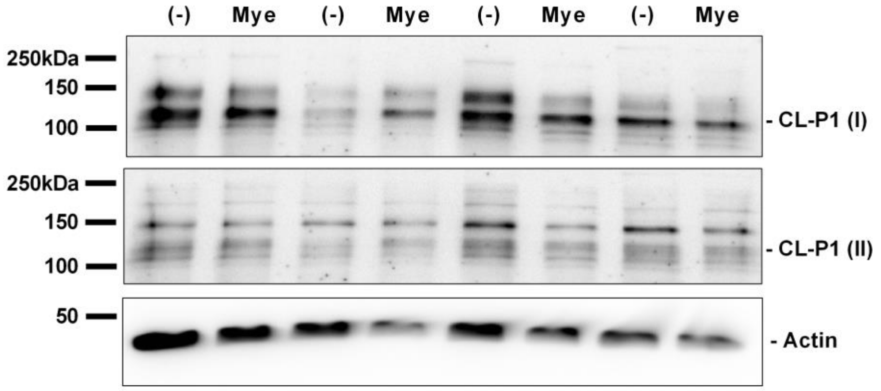

Figure s1*:* Myelin uptake increases the expression of CL-P1 in human monocytes (raw data). Human monocytes (hMono, n=5), were cultured with or without 100 µg/ml myelin for 24h. Western blot analysis was used to define CL-P1 expression. Two antibodies were used to define CL-P1 expression.

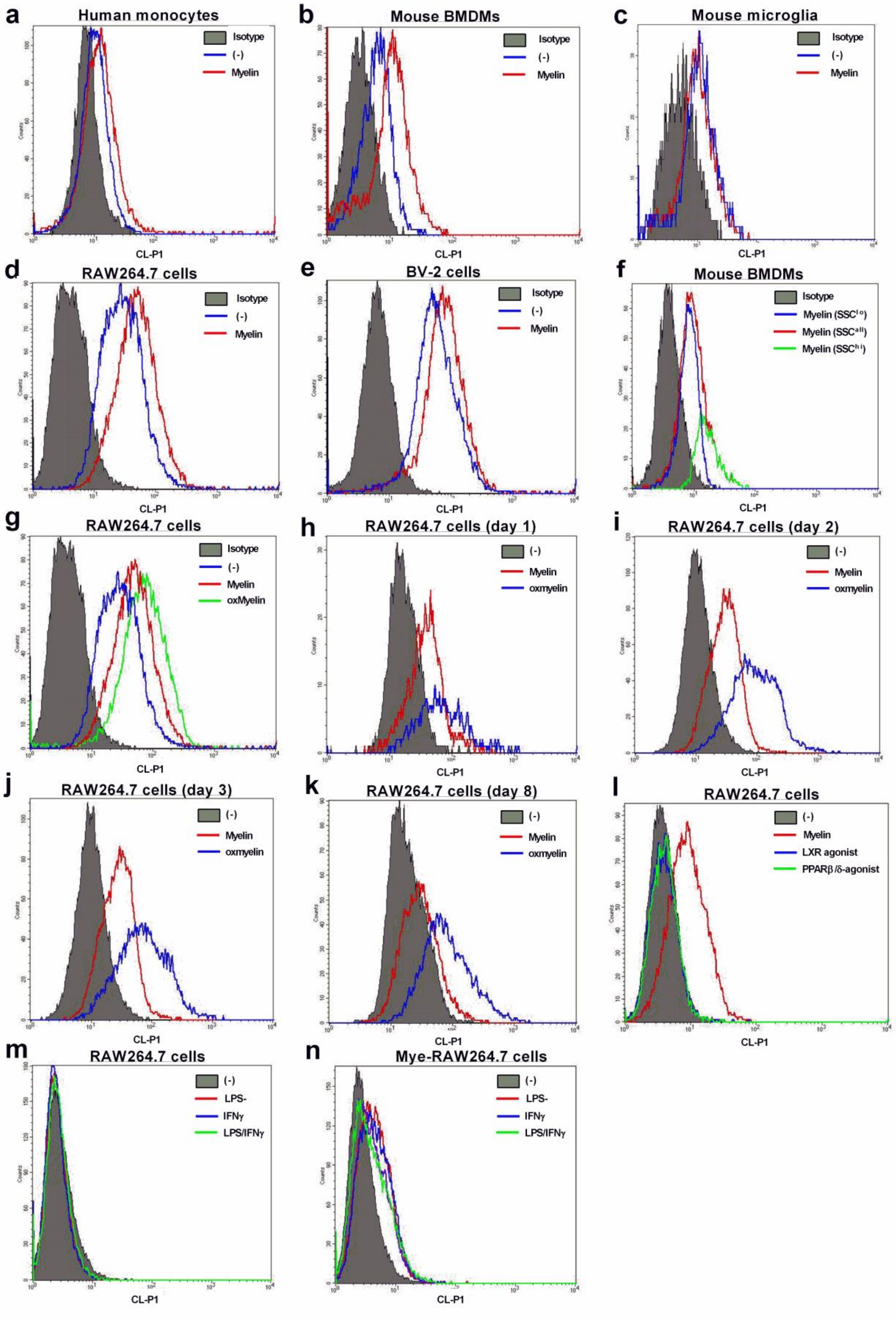
a
Human monocytes
Isotype
(-)
Myelin
CL-P1
b
Mouse BMDMs
Isotype
(-)
Myelin
CL-P1
c
Mouse microglia
Isotype
(-)
Myelin
CL-P1
d
RAW264.7 cells
Isotype
(-)
Myelin
CL-P1
e
BV-2 cells
Isotype
(-)
Myelin
CL-P1
f
Mouse BMDMs
Isotype
Myelin (SSC^lo)
Myelin (SSC^all)
Myelin (SSC^hi)
CL-P1
g
RAW264.7 cells
Isotype
(-)
Myelin
oxMyelin
CL-P1
h
RAW264.7 cells (day 1)
(-)
Myelin
oxmyelin
CL-P1
i
RAW264.7 cells (day 2)
(-)
Myelin
oxmyelin
CL-P1
j
RAW264.7 cells (day 3)
(-)
Myelin
oxmyelin
CL-P1
k
RAW264.7 cells (day 8)
(-)
Myelin
oxmyelin
CL-P1
l
RAW264.7 cells
(-)
Myelin
LXR agonist
PPARβ/δ-agonist
CL-P1
m
RAW264.7 cells
(-)
LPS-
IFNγ
LPS/IFNγ
CL-P1
n
Mye-RAW264.7 cells
(-)
LPS-
IFNγ
LPS/IFNγ
CL-P1

Figure s2*: *Myelin uptake increases the surface expression of CL-P1 on myeloid cells (raw data). (a-e) Primary human monocytes (a), primary mouse BMDMs (b) and microglia (c), RAW264.7 cells (d), and BV-2 cells (e) were cultured with or without 100 µg/ml myelin for 24h, after which CL-P1 expression was determined with flow cytometry. (f) Mouse BMDMs cultured with 100 µg/ml myelin for 24h. CL-P1 expression was determined in high granular (SSC^{hi}), low granular (SSC^{lo}), and all cells (SSC^{all}) by using flow cytometry. (g) RAW264.7 cells were exposed to 100 µg/ml unmodified and CU^2-oxidized myelin for 24h, after which CL-P1 expression was determined. (h-k) RAW264.7 cells were cultured with 100 µg/ml unmodified or CU^2-oxidized myelin or left untreated for 1 (h), 2 (i), 3 (j), and 8 days (k). CL-P1 expression was determined by using flow cytometry. (l) RAW264.7 cells were cultured with a 1 µM T0901317 (LXR agonist), 1 µM GW501516 (PPARβ/δ agonist), or 100 µg/ml myelin for 24h, after which CL-P1 expression was determined with flow cytometry. (m,n) Untreated (m) or myelin treated RAW264.7 cells (n) were exposed to 500 U/ml IFNγ, 100 ng/ml LPS, a combination 500 U/ml IFNγ and 100 ng/ml LPS, or left untreated. CL-P1 expression was determined by using flow cytometry.

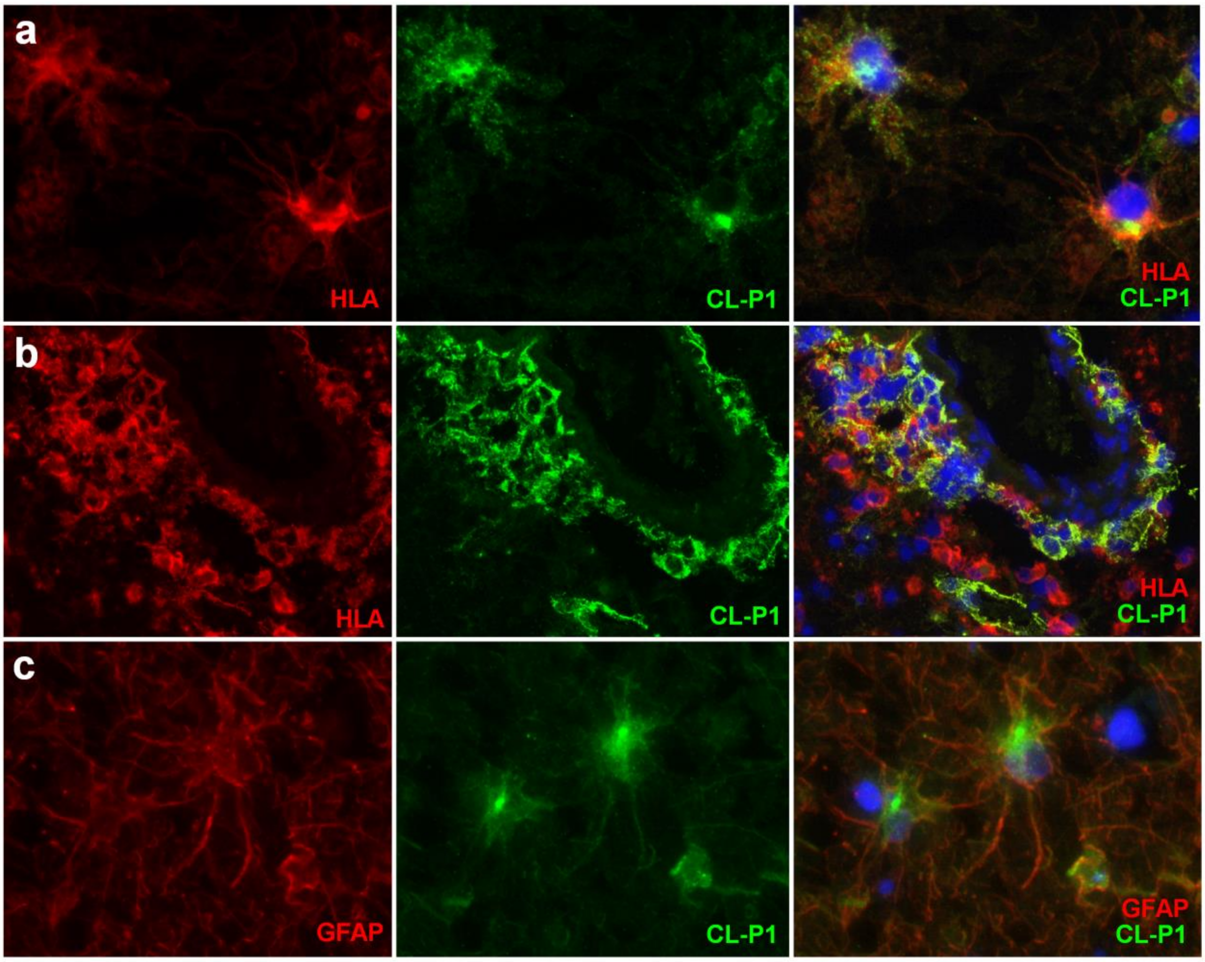

Figure s3*:* CL-P1 is expressed by phagocytes and astrocytes in MS lesions. (a-c) Representative images of active MS lesion stained for CL-P1 (R&D) and HLA-DR (a, 100x magnification; b, 40x magnification), and CL-P1 (R&D) and GFAP (c, 100x magnification).

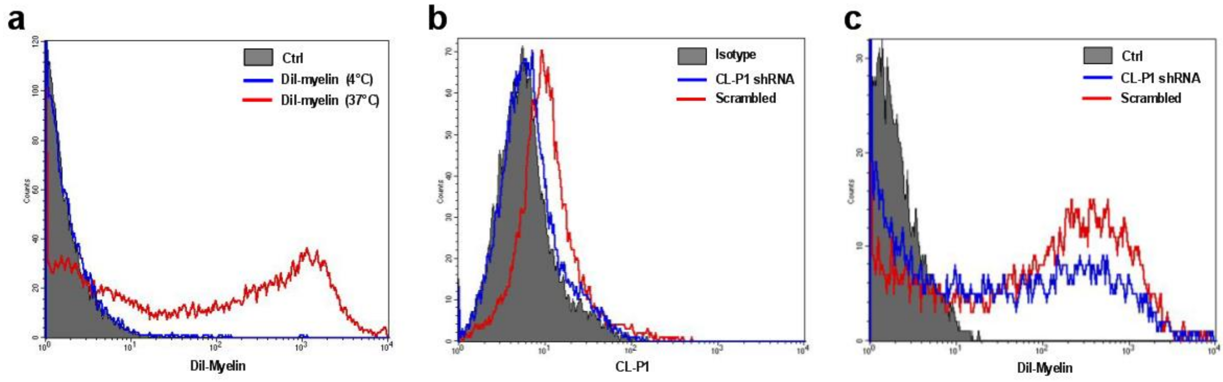

Figure s4: CL-P1 is involved in the uptake of myelin (raw data). (a) HEK293.1 cells were exposed to DiI-labeled myelin for 1.5h (n=4). Myelin uptake was assessed using flow cytometry. Cells were exposed to myelin at 4°C (binding) or 37°C (binding and uptake). (b) HEK293.1 cells were exposed to scrambled shRNA or a pool of shRNA directed against CL-P1 (shRNA1-4) for 48h. The mRNA and protein expression of CL-P1 was determined using flow cytometry. (c) HEK293.1 cells were exposed to scrambled shRNA or a pool of shRNA directed against CL-P1 (shRNA1-4) for 48h. Next, DiI-labeled myelin was added for 1.5h. Flow cytometry was used to define myelin uptake.

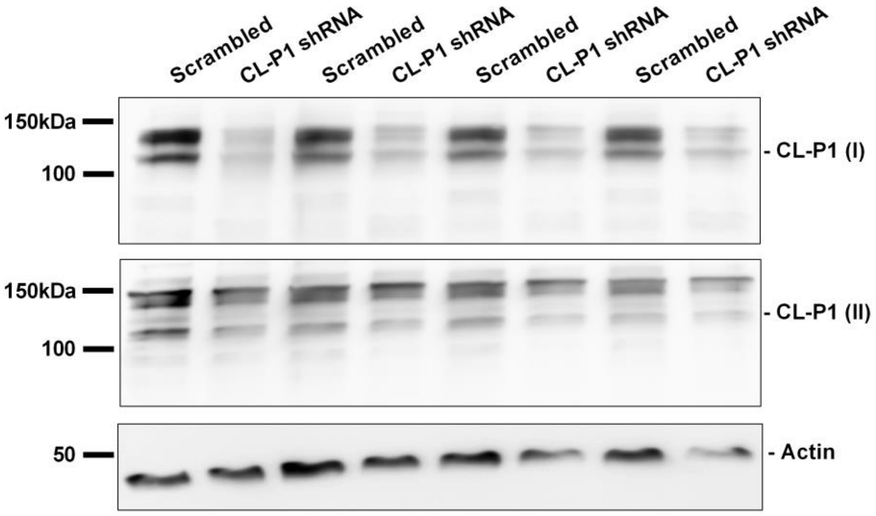

Figure s5: CL-P1 is involved in the uptake of myelin (raw data). HEK293.1 cells were exposed to scrambled shRNA or a pool of shRNA directed against CL-P1 (shRNA1-4) for 48h. The protein expression of CL-P1 was determined using western blot. Two antibodies were used to define CL-P1 expression.
